# Supplementary material for: Liquid biopsy-based single-cell metabolic phenotyping of lung cancer patients for informative diagnostics
Source: Nat Commun. 2019 Aug 26;10:3856. doi: 10.1038/s41467-019-11808-3 (PMC6710267; doi:10.1038/s41467-019-11808-3)
Supplement: Supplementary file 1 — Supplementary Information [file 41467_2019_11808_MOESM1_ESM.pdf]

Supplementary Materials

Liquid biopsy-based single-cell metabolic phenotyping of lung cancer patients for informative diagnostics

Li et al.,

Supplementary Figures

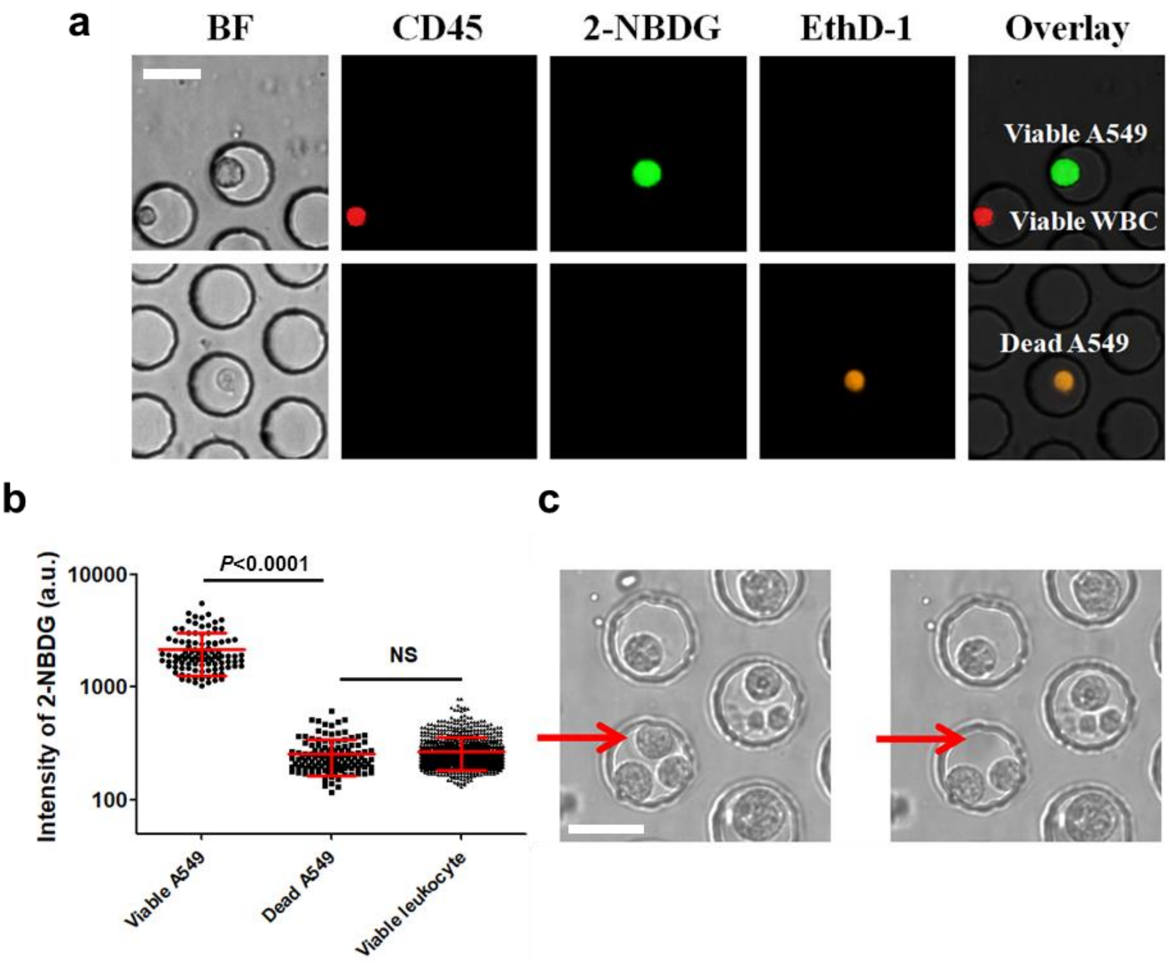

Supplementary Fig. 1 a Fluorescent images of a viable A549 cell, a viable leukocyte and a dead A549 cell (scale bar, 30 μm). **b** 2-NBDG uptake of viable leukocytes (n=1011), viable A549 cells (n=101), and dead A549 cells (n=124). Dead cells have minimal unspecific 2-NBDG signal (mean ± SD). **c** Precise single-cell retrieval from a microwell containing multiple cells. Left, before single cell retrieval; Right, after single cell retrieval (scale bar, 30 μm). Source data are provided as a Source Data file.

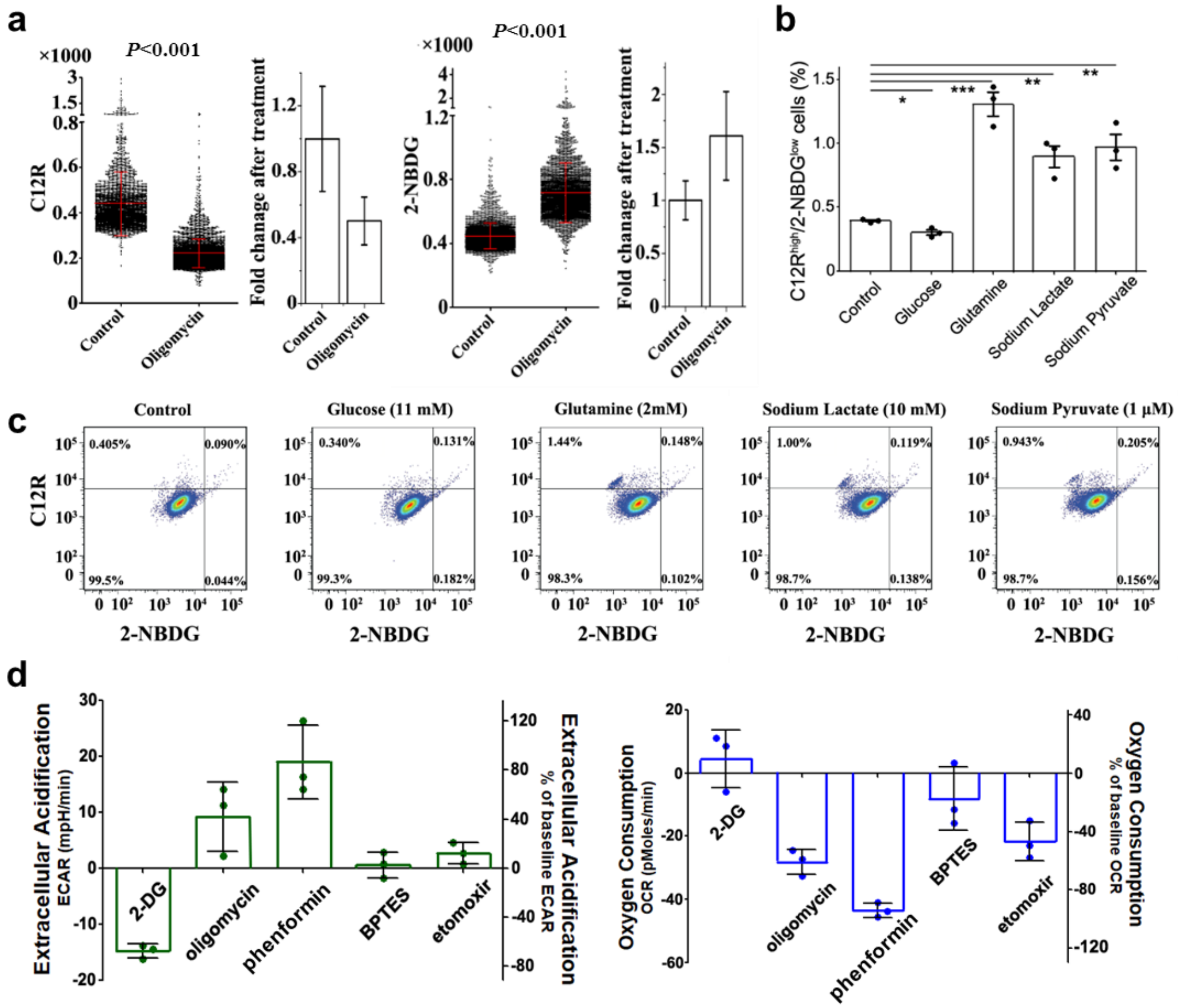

Supplementary Fig. 2 a On-chip metabolic cytometry of C12R signal (left, n=3329 for control, n=3674 for oligomycin) and 2-NBDG (right, n=3784 for control and n=3499 for oligomycin) uptake of A549 cells treated with DMSO control and oligomycin. The data are represented as scatter plots and bar columns (mean ± SD). **b** Percentages of C12R^{high}/2-NBDG^{low} cells in five perturbed conditions (vehicle, glucose, glutamine, sodium lactate or sodium pyruvate) are represents in bar columns (n=3 independent experiments, mean ± SEM), derived from three independent experiments. * $P < 0.05$; ** $P < 0.01$; *** $P < 0.001$. **c** Representative flow cytometry plots report 2-NBDG and C12R fluorescence intensity of A549 cells treated with vehicle, glucose, glutamine, sodium lactate or sodium pyruvate, respectively. It is noted that gating of C12R^{high}/2-NBDG^{low} cells shown in this figure is different from that of metabolically active cells. **d** Changes of ECAR and OCR of A549 cells in response to 2-DG (5 mM), oligomycin (2 μM), phenformin (25 μM), BPETS (10 μM), and etomoxir (200 μM) by using DMSO as the control (n=3 independent samples for

each condition). Each sample represented the average of ten cycles of ECAR and OCR measurements (mean ± SD). Source data are provided as a Source Data file.

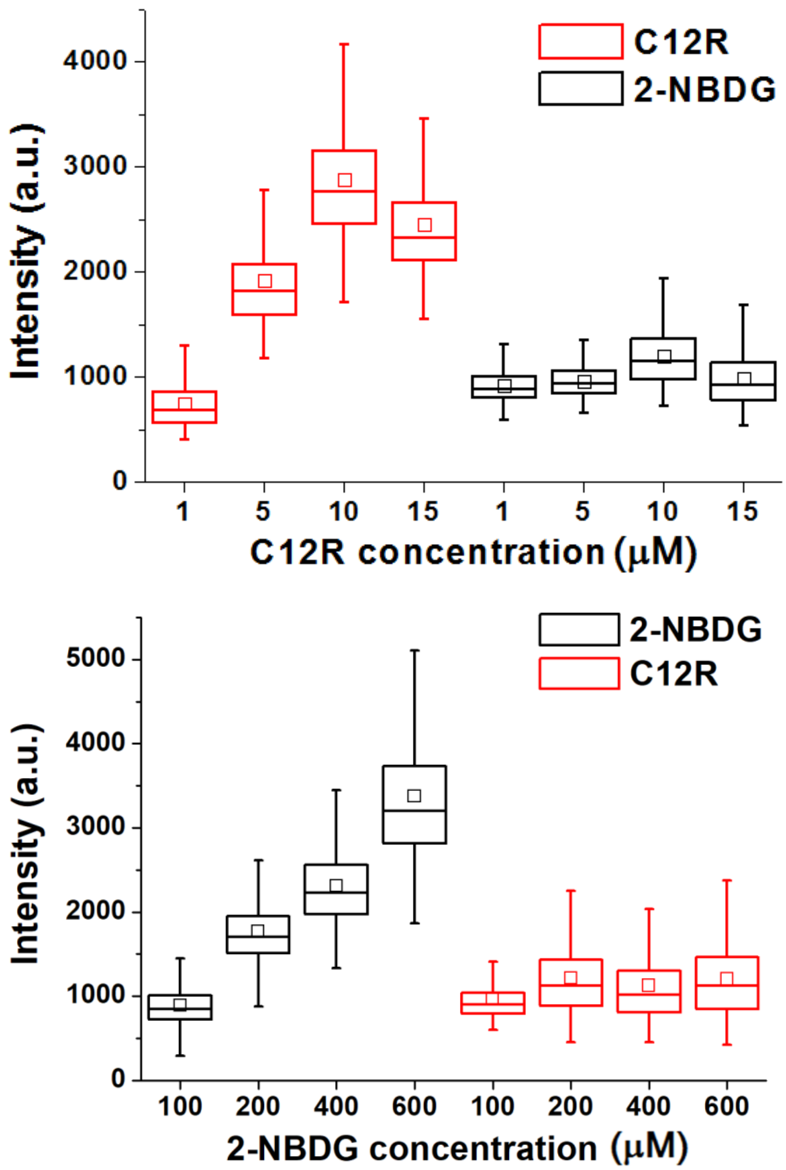

Supplementary Fig. 3. Validation of mutual interference of the two metabolic probes. Top, box chart of fluorescence intensity of C12R and 2-NBDG uptake in A549 cells under variable C12R concentrations (1 μM, 5 μM, 10 μM and 15 μM) and a fixed 2-NBDG concentration (100 μM) by on-chip metabolic cytometry; Bottom, box chart of fluorescence intensity of C12R and 2-NBDG uptake in A549 cells under variable 2-NBDG concentrations (100 μM, 200 μM, 400 μM and 600 μM) and a fixed C12R concentration (1 μM) by on-chip metabolic cytometry. The data was generated from single-cell measurements for each condition (Top, left to right, n=1030, 960, 1110, 941, 1036, 987,

1096, 931; Bottom, left to right, n=6773, 7401, 6886, 7078, 6862, 7267, 6841, 7058) and represented in box plots (center line, median; square, mean; box limits, upper and lower quartiles; whiskers, 1.5*inter-quartile range). Source data are provided as a Source Data file.

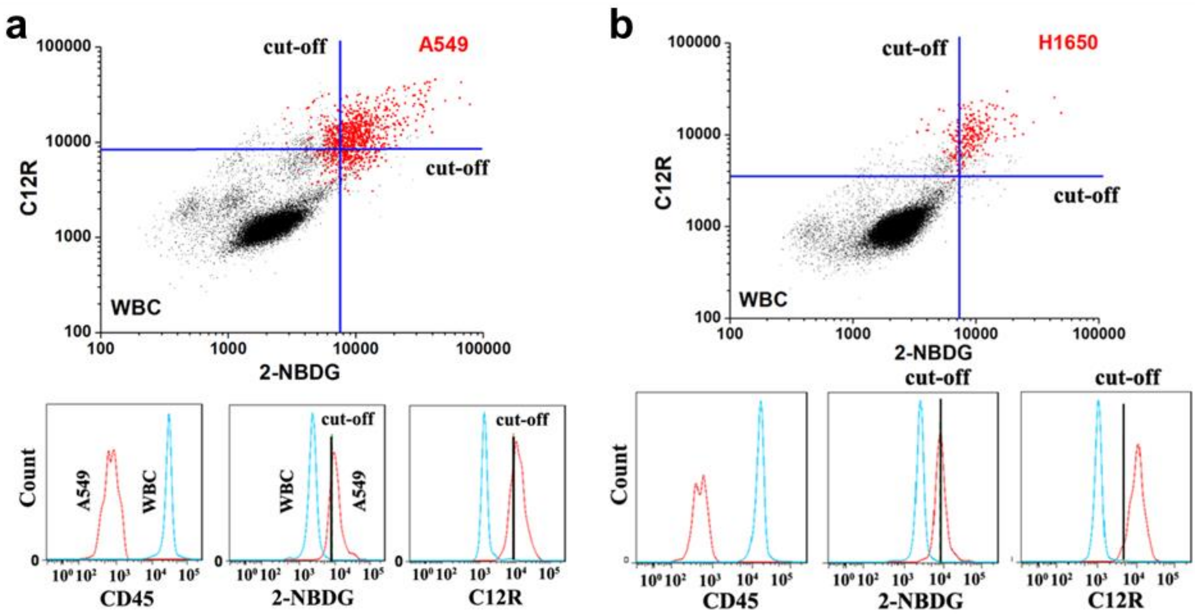

Supplementary Fig. 4 Cut-off determination for separating putative metabolically active tumor cells from leukocytes. Top, scatter plot generated from flow cytometry reports 2-NBDG and C12R fluorescence intensity of leukocytes spiked with (**a**) A549 and (**b**) H1650 cells. Leukocytes were isolated from the blood of a healthy donor. Bottom, histograms of CD45, 2-NBDG and C12R levels of leukocytes and spiked tumor cells. The cut-off values of metabolic markers 2-NBDG and C12R is defined as mean plus five standard deviations of leukocytes, as indicated by the black lines.

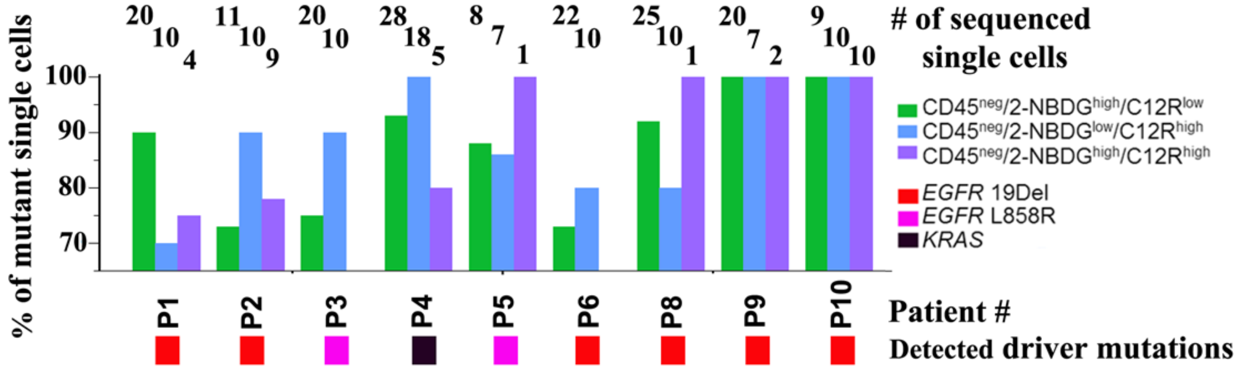

Supplementary Fig. 5 Percentage of single cells harboring oncogenic driver mutations that determine the malignancy of metabolically active cells. The detected oncogenic driver mutations were consistent with those detected in cell blocks of MPE or primary tumor sites

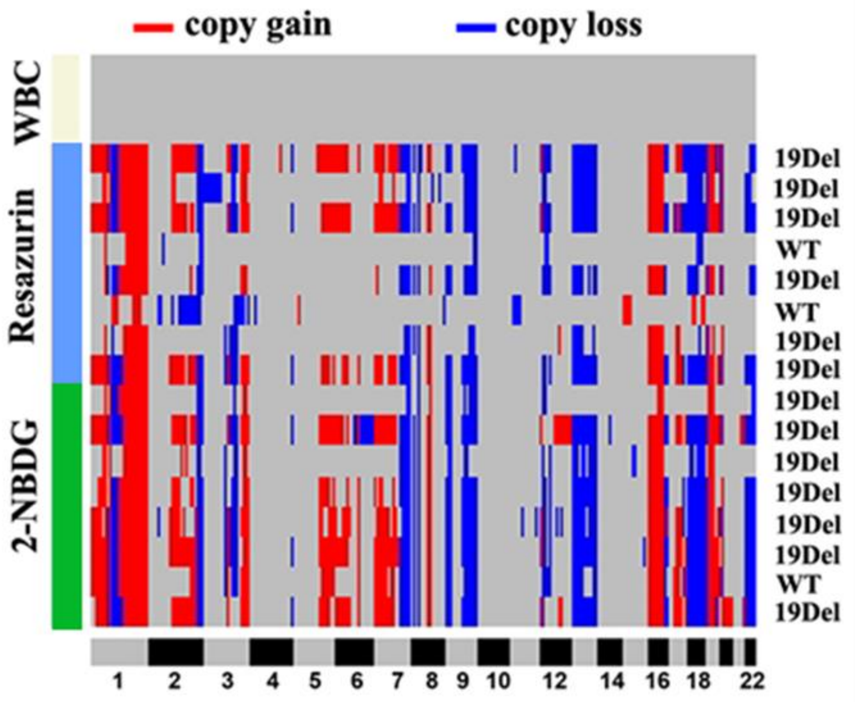

Supplementary Fig. 6 Single-cell CNV profiles and detected *EGFR* mutation status of metabolically active cells and white blood cells from P1.

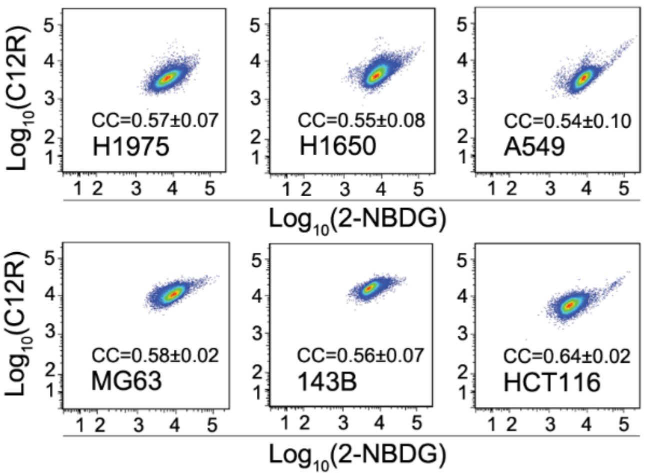

Supplementary Fig. 7 Representative flow cytometry plots report 2-NBDG and C12R fluorescence signals of H1975, H1650, A549, MG63, 143B and HCT116 cells that were assayed by 600 μM 2-NBDG and 1 μM C12R. 50,000 events were recorded for each sample. The correlation coefficients (CC) between 2-NBDG and C12R (mean ± SD), derived from four independent experiments, in these cell lines range from 0.54 to 0.64.

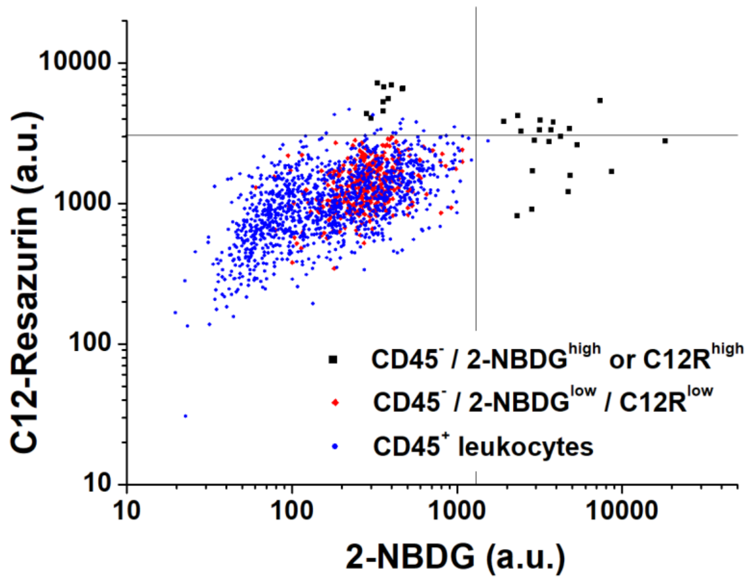

Supplementary Fig. 8 Scatter plot of OMC assays on Patient 2 MPE samples. 2-NBDG and C12R fluorescence intensity of all CD45 negative cells (black and red dots) and a portion of CD45 positive cells (blue dots) are shown. The cut-offs for identification of 2-NBDG^{high} and C12R^{high} cells (black dots) are shown in the figure as the black lines. 2-NBDG^{high} and C12R^{high} cells are candidate tumor cells and gated out by five and three standard deviations above mean of CD45^{+} leukocytes, respectively. Red dots represent CD45^{neg}/2-NBDG^{low}/C12R^{low} cells and CD45^{pos} leukocytes are displayed in blue dots. In Patient #2, 11 CD45^{neg}/2-NBDG^{high}/C12R^{low} cells, 10 CD45^{neg}/2-NBDG^{low}/C12R^{high} cells and 9 CD45^{neg}/2-NBDG^{high}/C12R^{high} cells were identified as candidate tumor cells that were found to harbor an *EGFR* 19Del (p. L747_E749del) and an A750P mutation in exon 19 of *EGFR*.

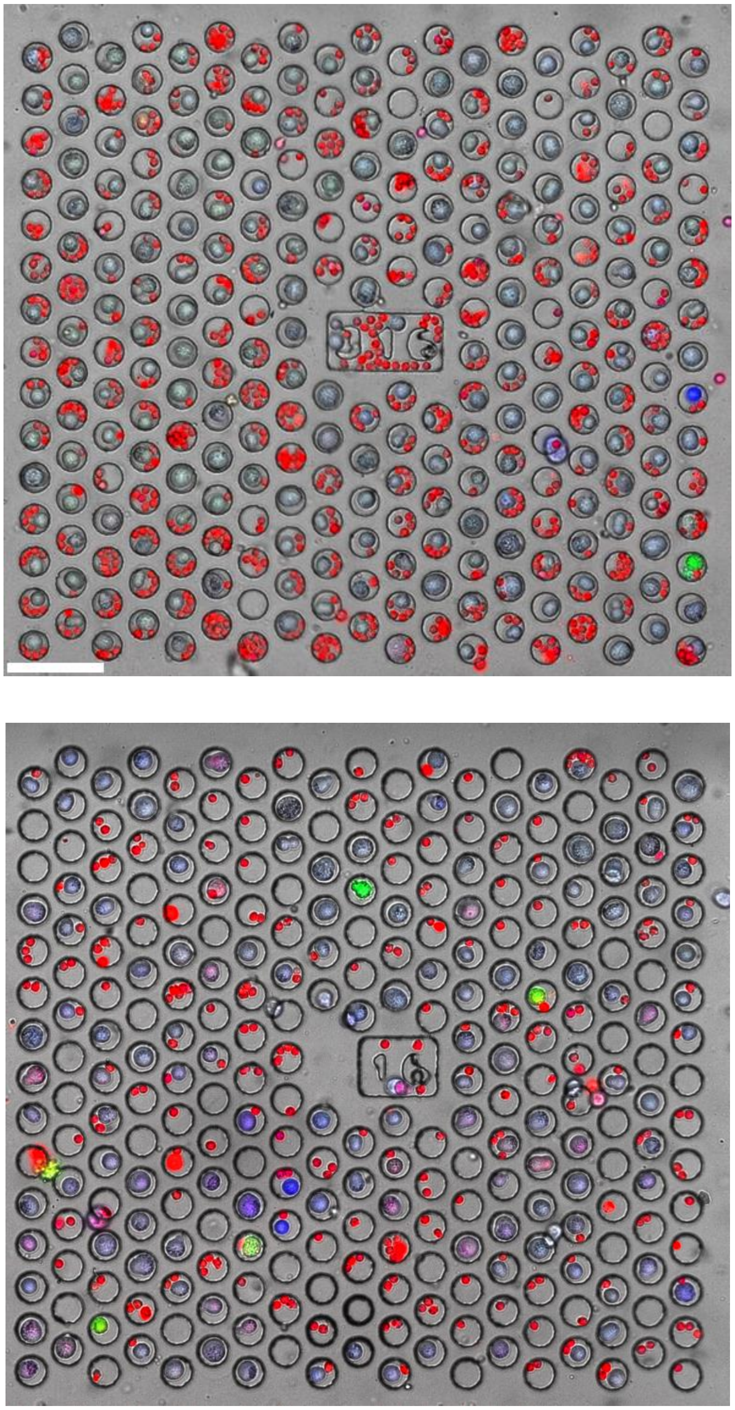

Supplementary Fig. 9 The bright field and fluorescence composite images of representative blocks shows both C12R^{high} cells and 2-NBDG^{high} cells. The fluorescence signals of CD45, 2-NBDG and C12R are shown in red, green and blue, respectively (scale bar, 100 μm).

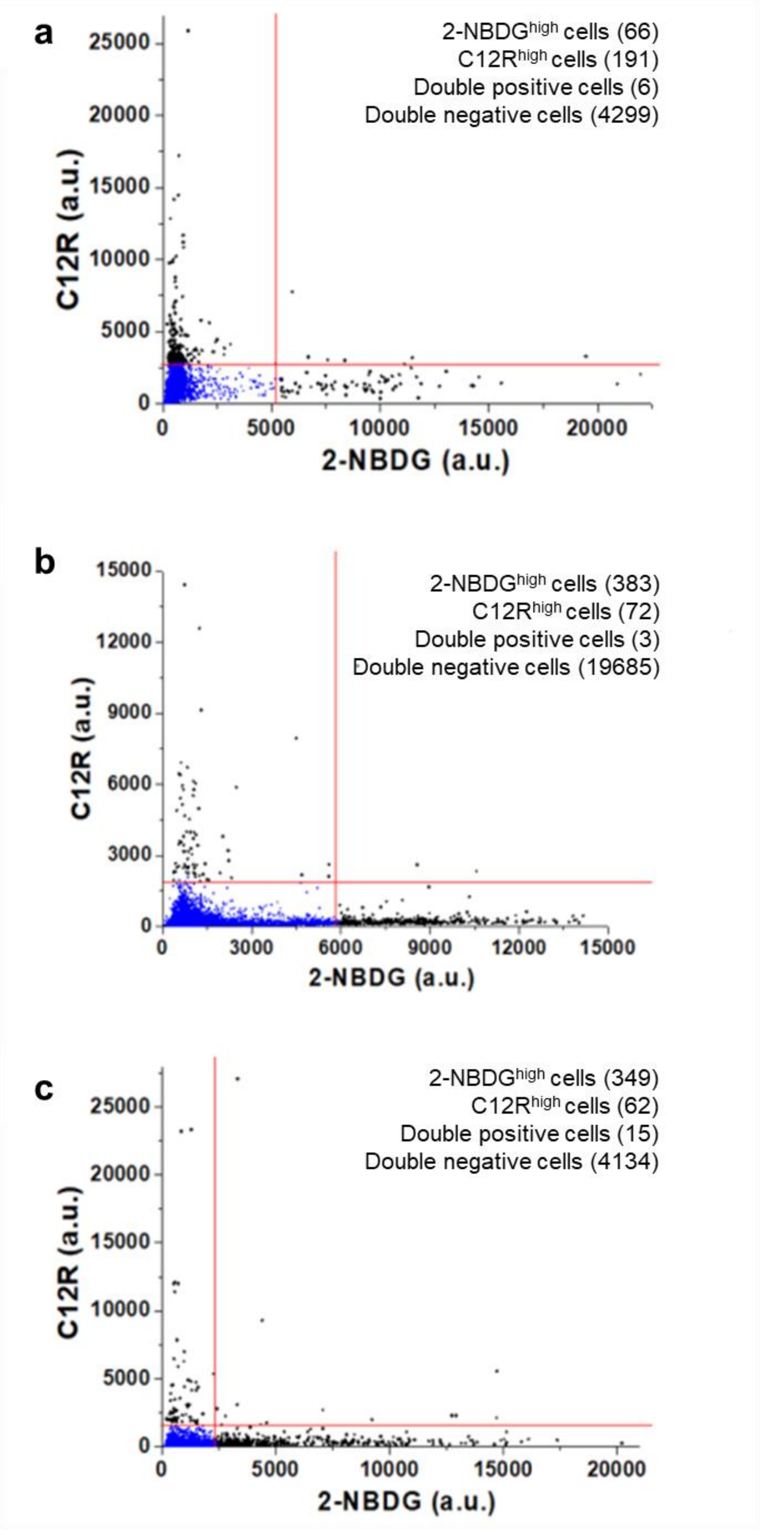

Supplementary Fig. 10 a-c Single-cell metabolic phenotyping of CD45 negative cells isolated from lung adenocarcinoma surgical resection tissues of three patients. Most of the metabolically active cells are differentially engaged in glycolysis or mitochondrial oxidation with very few double positive cells.

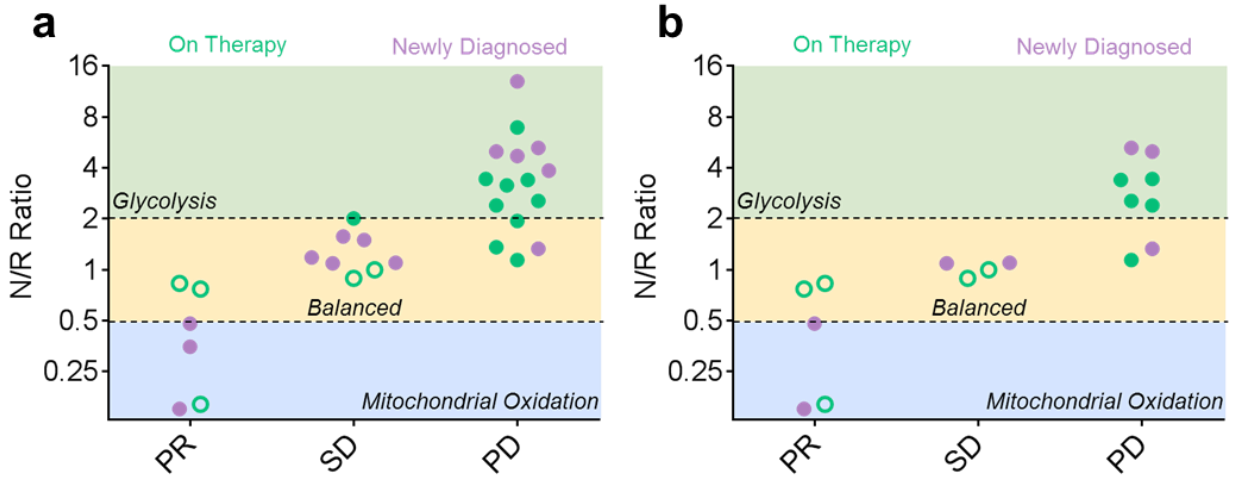

Supplementary Fig. 11 a,b N/R ratios calculated by metabolic phenotyping of MPE samples can predict patient therapy response profiles evaluated by RECIST criteria upon follow-up for all the patients **a** and for *EGFR*-mutant patient **b**. The different metabolic phenotypes of MPE samples that are classified by the boundary lines at N/R = 0.5 and 2 can segregate patient response. The green dots denote patients who were on therapies at the MPE draw and metabolic phenotyping. The orchid dots denote newly diagnosed patients who were receiving first-line therapies after the MPE draw and metabolic phenotyping. The blank green dots represent the patients with a PR response to the current therapy at the time of MPE draw and the solid green dots represent those with a SD or PD response at the time of MPE draw. PR: partial response, SD: stable disease, PD: progressive disease.

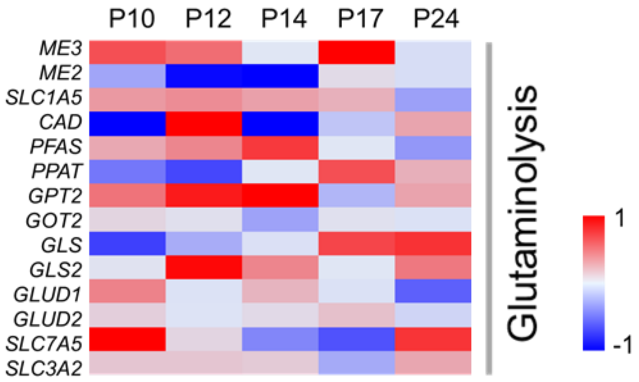

Supplementary Fig. 12 Log2 fold change of the expression levels of presentative genes involved in glutaminolysis between the two metabolic phenotypes (2-NBDG^{high} *vs* C12R^{high}) across 5 patients. The glutaminolysis-related genes showed a mixed pattern of change across patients.

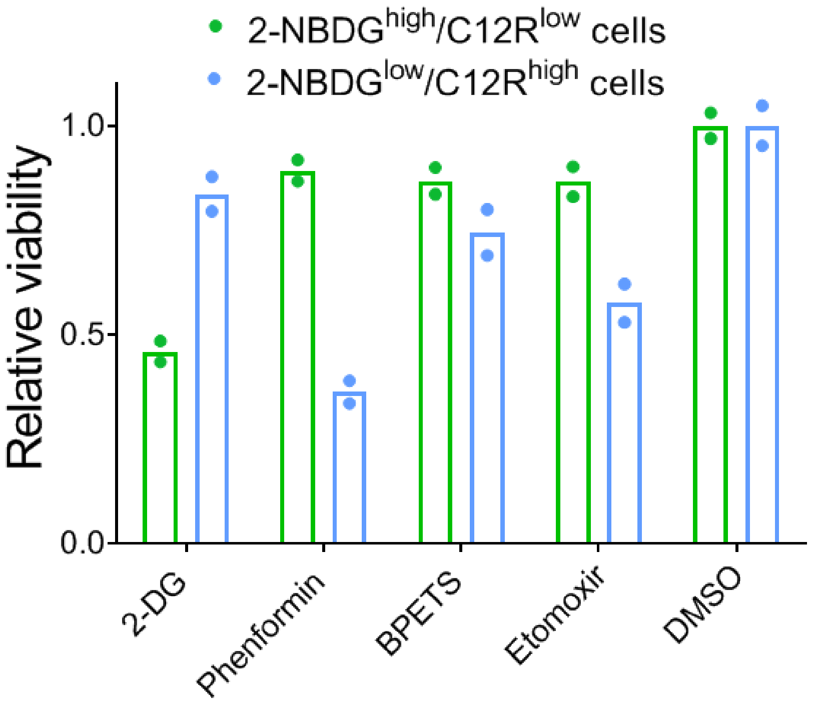

Supplementary Fig. 13 Relative viability of two metabolically active cell populations in response to 2-DG (5 mM), phenformin (25 μM), BPETS (10 μM), and etomoxir (200 μM) , respectively, with respect to the DMSO control (* $P<0.05$; ** $P<0.005$; NS, not significant). Data are presented as the mean ± SD (n=2 independent samples for each condition). Source data are provided as a Source Data file.

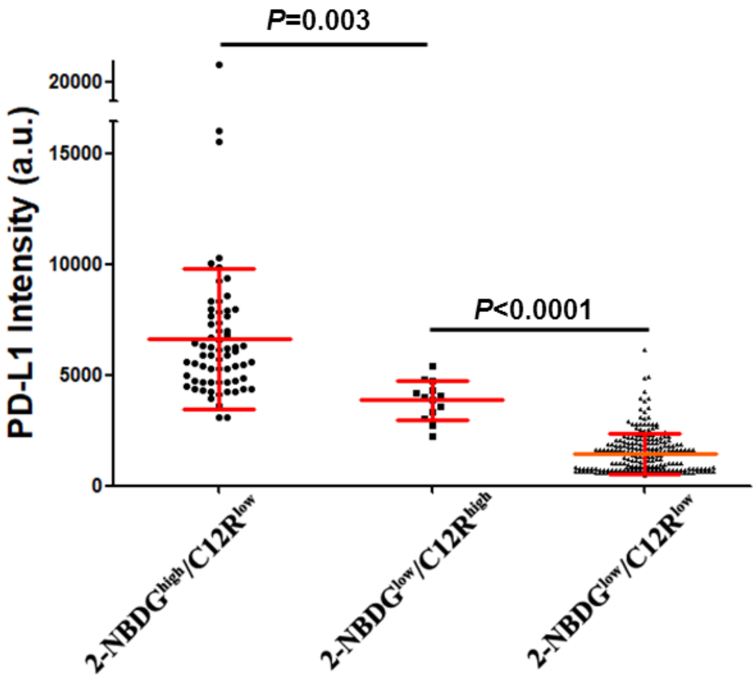

Supplementary Fig. 14 PD-L1 expression levels, quantified by the single-cell immunofluorescence staining, for the three metabolic phenotypes of $CD45^{neg}$ cells from MPE sample of P31. All $2\text{-NBDG}^{high}/C12^{high}$ cells (n=66) and $2\text{-NBDG}^{low}/C12R^{high}$ cells (n=13) on a chip as well as randomly selected double negative cells (n=244) were assayed and shown in the figure (mean ± SD). Source data are provided as a Source Data file.

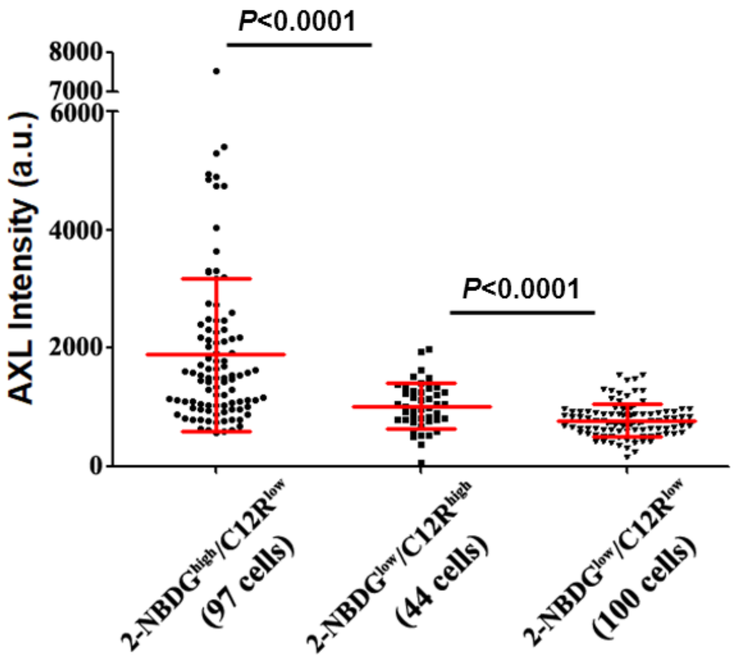

Supplementary Fig. 15 AXL fluorescence intensity of the three metabolic phenotypes of CD45^{neg} cells from MPE sample of P29. All 2-NBDG^{high} cells (n=97) and C12R^{high} (n=44) as well as randomly selected double negative cells (n=100) were assayed and shown in the figure (mean ± SD). Source data are provided as a Source Data file.

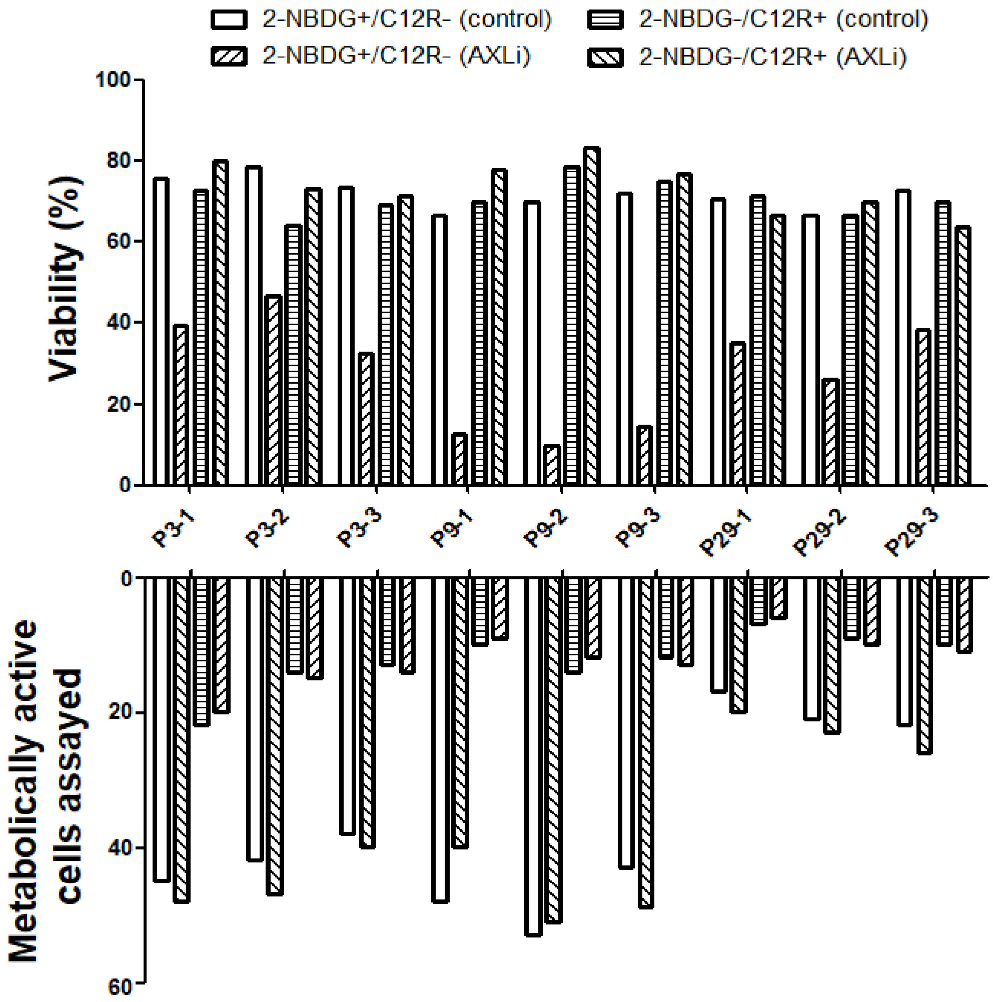

Supplementary Fig. 16 The number and viability of metabolically active cells assayed under DMSO control and AXL inhibition treatment in P3, P9 and P29. Each experiment had three replicates.

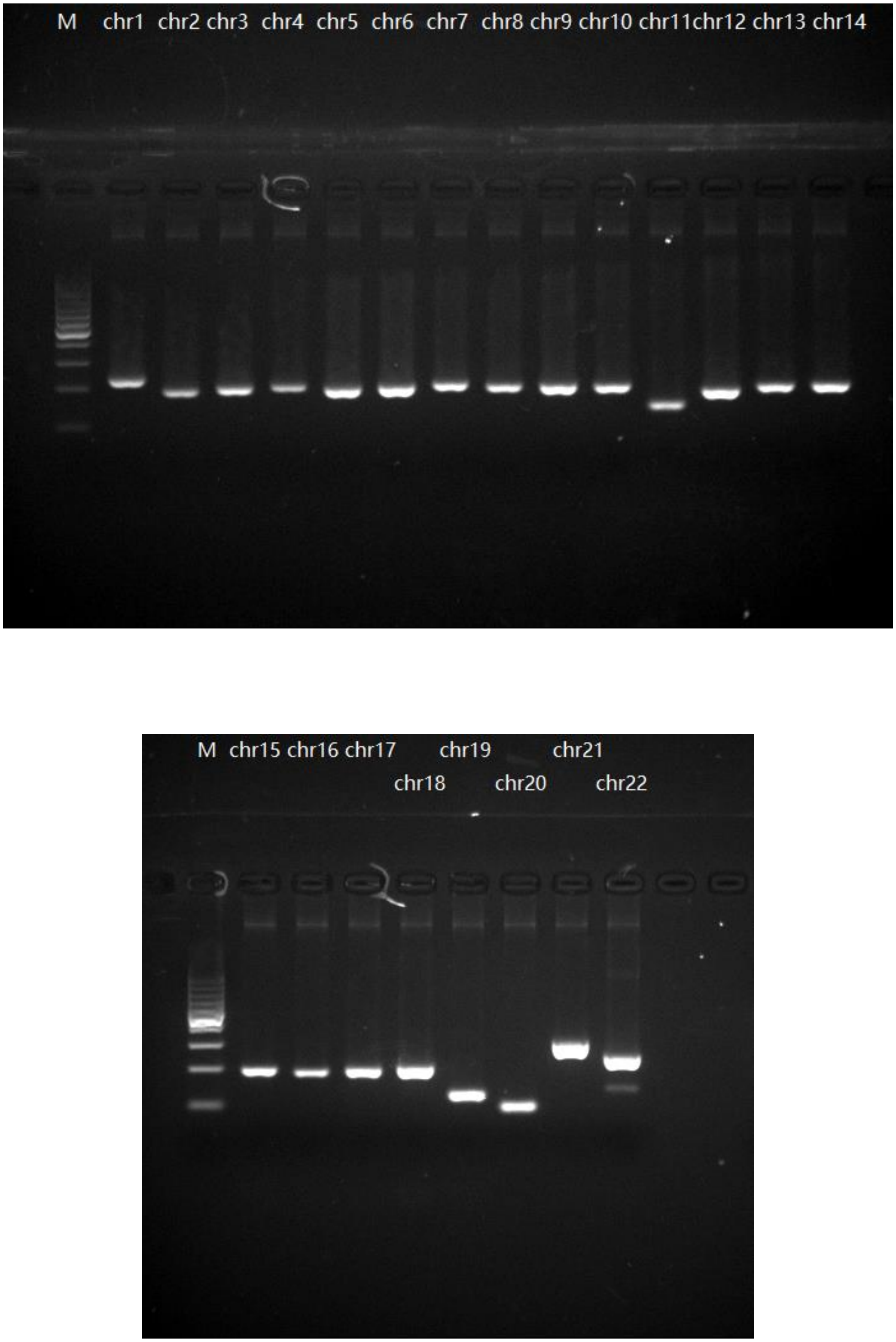

Supplementary Fig. 17. Evaluation of WGA efficiency using specific primers for 22 chromosomes (see Supplementary Table 5). 22 PCR reactions were performed and products of PCR reactions were separated by gel electrophoresis.

Supplementary Tables

Supplementary Table 1 Metabolic phenotype, treatment history and other clinical metadata of LADC patients[a].

No.	Metabolic phenotype[b]	Treatment before PE draw	Treatment at or after PE draw[c]	ECOG at PE draw	Response at PE draw	ECOG at follow-up	Response at follow-up	Survival (months)
1	MO (0.48)	None	1st line: Gefitinib	1	N/A	0	PR	20.6 (alive)
2	B (1.10)	None	1st line: Pemtrexed+Carboplatin+Avastin	1	N/A	1	SD	19.6 (alive)
3	G (2.40)	1st line: Icotinib; 2nd line: Pemtrexed+Carboplatin	3rd line: Osimetrinib*; 4th line: Pemtrexed+Avastin	1	SD	2	PD	15.6
4	G (6.94)	1st line: Pemtrexed	2nd line: Vinorelbine*	1	PD	5	Dead	0.8
5	B (1.14)	1st line: Docetaxol+Carboplatin; 2nd line: Gefitinib	3rd line: Osimertinib*	1	SD	5	Dead	7.13
6	B (0.71)	None	1st line: Traditional Chinese Medicine*	1	N/A	2	PD	9.1
7	G (3.40)	None	1st line: Pemtrexed+Carboplatin*; 2nd line: Gefitinib; 3rd line: Docetaxol	0	PD	1	PD	10.1
8	G (2.55)	None	1st line: Gefitinib*; 2nd line: Pemtrexed+Carboplatin	1	PD	5	Dead	7.03
9	G (5.00)	None	1st line: Gefitinib	2	N/A	5	Dead	1.93
10	MO (0.15)	None	1st line: Gefinitib+Furuquitinib	1	N/A	0	PR	15.2 (alive)
11	B (1.33)	None	1st line: Pemtrexed+Carboplatin	2	N/A	5	Dead	7.23
12	G (3.44)	1st line: Pemtrexed+Carboplatin; 2nd line: Icotinib; 3rd line:	5th line: Osimetrinib*	1	SD	5	Dead	4.03

		Pemtrexed+Avastin; 4th line: Avastin						
13	B (0.83)	1st line: Pemtrexed+Carboplatin+Avastin	2nd line: Gefitinib	1	PR	1	PR	16.0 (alive)
14	B (1.36)	1st line: Gefitinib; 2nd line: Osimertinib	3rd line: Tegafur Gimeracil Oteracil Potassium Capsule*	2	PD	5	Dead	7.13
15	MO (0.35)	None	1st line: Crizotinib	1	N/A	0	PR	15.9 (alive)
16	G (13.00)	None	1st line: Pemtrexed+Carboplatin	2	N/A	5	Dead	2.43
17	MO (0.16)	None	1st line: Gefinitib+Furuquitinib*	1	PR	0	PR	14.8 (alive)
18	B (1.09)	None	1st line: Icotinib	1	N/A	1	SD	20.0 (alive)
19	B (0.89)	1st line: Pemtrexed+Carboplatin	2nd line: Gefitinib*	1	PR	1	SD	17.7 (alive)
20	G (3.86)	None	1st line: Gemcitabine+Carboplatin	2	N/A	5	Dead	3.03
21	MO (0.12)	None	No anti-cancer treatment	2		5	Dead	3.03
22	B (1.50)	None	1st line: Pemtrexed+Carboplatin	2	N/A	2	SD	15.4 (alive)
23	B (1.57)	None	1st line: Pemtrexed+Carboplatin	1	N/A	1	SD	10.3
24	B (1.94)	Adjuvant: Navelbine+Carboplatin; 1st line: Gem+Carboplatin; 2nd line: Pemtrexed+Carboplatin; 3nd line: Fruquintinib	4th line: Tegafur Gimeracil Oteracil Potassium Capsule	1	SD	5	Dead	5.93
25	B (1.18)	None	1st line: Pemtrexed+Carboplatin	1	N/A	1	SD	14.3 (alive)
26	B (1.00)	Adjuvant: Navelbine+Carboplatin; 1 st line: Gefitinib	2nd line: Osimertinib	1	PR	1	SD	14.3 (alive)
27	G (3.15)	None	1st line: Crizotinib*	1	SD	5	Dead	5.5
28	B (0.77)	1st line: Pemtrexed+Carboplatin	2nd line: Gefitinib*	1	PR	1	PR	5.9 (alive)
29	G (2.01)	None	1st line: Pemtrexed+Carboplatin*	1	SD	1	SD	6.0 (alive)
30	G (4.70)	None	1st line: Gemcitabine+Carboplatin	1	N/A	5	Dead	3.6

31	G (5.25)	None	1st line: Gefitinib	2	N/A	5	Dead	1.3
32	R (0.04)	None	N/A[d]	1	N/A	N/A[d]	N/A[d]	N/A[d]

[a]Newly diagnosed patients are highlighted in grey. For P2, while the OMC assay identified *EGFR*^{19Del} driver oncogene mutation in the MPE, the clinical standard cell block analysis failed to identify any driver mutations due to the low abundance of tumor cells in the MPE. P2 was therefore treated as wild type patients with chemotherapy. P6 was not receiving appropriate anti-tumor treatment after diagnosis due to financial reasons. He instead only received palliative care with traditional Chinese medicine. P16 bearing *ROS1* gene alteration was receiving the 1st line treatment before the approval of crizotinib by China FDA (September 2017). He therefore received 1st line chemotherapy treatment. P21 had an underdetermined pathological diagnosis and thus did not receive any clinical treatment. [b]Key for metabolic phenotypes: G, glycolysis (N/R≥2); B, balanced (0.5<N/R<2); MO, mitochondrial oxidation (N/R≤0.5. [c]The * represents PE development in the treatment (not prior to the treatment). [d]This patient was most recently enrolled in this study and treatment-naïve.

Supplementary Table 2. **Primers used in this study for targeting oncogenic driver mutations. All primers were synthesized by Life Technologies.**

Name	Sequence
BRAF-15F	CTCATCCTAACACATTTCAAGCCC
BRAF-15R	CAGCATCTCAGGGCCAA
KRAS-2F	TGAGAGCCTTTAGCCGCC
KRAS-2R	TACCCTCTCACGAAACTCTG
EGFR-18F	TGGAGAAGCTCCCAACCAA
EGFR-18R	TTCCCAAACACTCAGTGAAACA
EGFR-19F	GTGGCACCATCTCACAATT
EGFR-19R	ATGCTCCAGGCTCACCAAG
EGFR-20F	CTTTATCCAATGTGCTCCTC
EGFR-20R	TCTCCCTTCCCTGATTACCT
EGFR-21F	TTCGCCAGCCATAAGTCCT
EGFR-21R	TCATTCACTGTCCCAGCAAG

Supplementary Table 3. Explanatory and observation variable matrices used in PLS-DA model[a].

Explanatory variables						Observation variables	
Patient	2-NBDG	C12R	DP	N/R	Metabolically active cells / 10 mL PE	RECIST	Response Classification
P1	20	42	4	0.48	30	PR	P
P2	11	10	9	1.1	61	SD	P
P3	36	15	0	2.4	11	PD	N
P4	125	18	5	6.94	37	Dead	N
P5	8	7	1	1.14	2	Dead	N
P7	34	10	3	3.4	40	PD	N
P8	51	20	1	2.55	185	Dead	N
P9	35	7	2	5	157	Dead	N
P10	9	59	17	0.15	79	PR	P
P11	12	9	2	1.33	3	Dead	N
P12	31	9	3	3.44	6	PD	N
P13	10	12	6	0.83	6	PR	P
P14	19	14	1	1.36	20	Dead	N
P15	16	46	24	0.35	430	PR	P
P16	26	2	18	13	41	Dead	N
P17	3	19	1	0.16	2	PR	P
P18	12	11	3	1.09	33	SD	P
P19	8	9	0	0.89	3	SD	P
P20	27	7	10	3.86	11	Dead	N
P22	15	10	2	1.5	17	SD	P
P23	11	7	7	1.57	3	SD	P
P24	33	17	18	1.94	309	PD	N
P25	26	22	9	1.18	1267	SD	P
P26	10	10	1	1	11	SD	P
P27	299	95	23	3.15	334	Dead	N
P28	37	48	15	0.77	3333	PR	P
P29	23	11	6	2.01	80	SD	P
P30	127	27	20	4.7	1933	Dead	N
P31	63	12	5	5.25	267	Dead	N

[a]Columns titled 2-NBDG and C12R list the number of $CD45^{neg}$, $2\text{-}NBDG^{high}$ and $C12R^{high}$ cells per 500,000 input cells, respectively. DP denotes the number of double positive cells

(CD45^{neg}/2-NBDG^{high}/C12R^{high}). Metabolically active cells include all the 2-NBDG^{high}, C12R^{high}, and double positive CD45^{neg} cells.

Supplementary Table 4. **Prior and posterior classification and scores using leave-one-out validation.**

Patient	Weight	Response	Predicted Response	P(N)	P(P)
P1	1	P	P	0.371	0.629
P2	1	P	P	0.423	0.577
P3	1	N	N	0.515	0.485
P4	1	N	N	0.804	0.196
P5	1	N	**P**	0.425	0.575
P7	1	N	N	0.563	0.437
P8	1	N	N	0.532	0.468
P9	1	N	N	0.636	0.364
P10	1	P	P	0.323	0.677
P11	1	N	**P**	0.438	0.562
P12	1	N	N	0.563	0.437
P13	1	P	P	0.408	0.592
P14	1	N	**P**	0.442	0.558
P15	1	P	P	0.344	0.656
P16	1	N	N	0.885	0.115
P17	1	P	P	0.361	0.639
P18	1	P	P	0.423	0.577
P19	1	P	P	0.412	0.588
P20	1	N	N	0.579	0.421
P22	1	P	P	0.448	0.552
P23	1	P	P	0.450	0.550
P24	1	N	**P**	0.478	0.522
P25	1	P	P	0.395	0.605
P26	1	P	P	0.418	0.582
P27	1	N	N	0.789	0.211
P28	1	P	P	0.302	0.698
P29	1	P	P	0.480	0.520
P30	1	N	N	0.672	0.328
P31	1	N	N	0.675	0.325

Supplementary Table 5. Primers used in this study for targeting 22 loci on different chromosomes. All primers were synthesized by Genewiz (Suzhou, China).

Name	Sequence
Chr1F	TTTAGGCGTCATCTGAGGGTA
Chr1R	TGGCAGCAGTATGGAGAATGTA
Chr2F	AGCGGGAGGGACTATTTCAC
Chr2R	GGATCGTTCAAAGGGAACTG
Chr3F	CCCTTGTACTGGCTCGTGTT
Chr3R	CTTGCACATGAAGGTCTGGA
Chr4F	GAGCATCTCTTGGCTCTGCT
Chr4R	TTGGGAAAGCACAGATCCTT
Chr5F	ACGGACAGTGGACAGATTGC
Chr5R	CCACTGTGCCACCCCATT
Chr6F	GAGGAGGGCAAGGAGAGAGT
Chr6R	ACCCTCCAGTGTGCAAAAAC
Chr7F	CTTCCTGCCATTCCACAAGT
Chr7R	CCCACTTTCATGCCTCTGAT
Chr8F	CTTCCCTGCCTTGCTCTCTA
Chr8R	CGGGACATTTCAGCAATCTT
Chr9F	CTGTGGAGCAGCTGTTTCTG
Chr9R	GAATTCACAAAGCCCCAAGA
Chr10F	CCCCTCATTCAAATCAGCAT
Chr10R	CAGGCAAAAGCTGGAGTTTC
Chr11F	TGAATGAGAACGCAGATGTGA
Chr11R	CACAAAGCATCCAGGGTCATT
Chr12F	ATCATGGAAATGCAGCCTCT
Chr12R	AGAACCCAGCTGGAATGATG
Chr13F	TGTTTCATGGAGTCCTGCTG
Chr13R	GGAGGCAAGAACCAAACAAA
Chr14F	AGCCAAGACGTACCCTCTCA
Chr14R	TGCTTTACACCAATCCCACA
Chr15F	TCAGCATGGGTTATGGGTTT
Chr15R	CCCAGATGATGGAGAGGAAA
Chr16F	GCCTGTGTTTGCTGATGAAA
Chr16R	GGGCAACGACCGTACTTAAA
Chr17F	TCCTGGGCTAGCCTTTTACA
Chr17R	ATCGCTTGAGCACTGAAGGT
Chr18F	AGACGAGCCTTTCTCTGTCG

Chr18R	TCGAGACCATCCCCACTAAC
Chr19F	AGTTGAGGAGATGGTGGAGC
Chr19R	AACAGGAGCCTTGGTCAGTC
Chr20F	CTGGTCAAACATCTCCCTCGT
Chr20R	CTCCACGCATCTTACATCACCT
Chr21F	GGACTTTGCTGACGGGATTA
Chr21R	GAACTAACGACCTCACGCTTG
Chr22F	TCCACAACCCCTTATCTTACCC
Chr22R	ACCTCAGGTGATCTACCCGC
