# Supplementary material for: Liquid biopsy-based single-cell metabolic phenotyping of lung cancer patients for informative diagnostics
Source: Nat Commun. 2019 Aug 26;10:3856. doi: 10.1038/s41467-019-11808-3 (PMC6710267; doi:10.1038/s41467-019-11808-3)
Supplement: Supplementary file 2 — Description of Additional Supplementary Files [file 41467_2019_11808_MOESM2_ESM.docx]

**Description of Additional Supplementary Files**

**File name: Supplementary Data 1 (Excel Spreadsheet)**

**Description:** RNA-seq data (in RPKM) for 5 randomly selected patients (P10, P12, P14, P17, P24)

**File name: Supplementary Data 2 (Excel Spreadsheet)**

**Description:** Single nucleotide variations (SNV) shared by a specific metabolic phenotype across at least 4 out of 5 patients.

**File name: Supplementary Data 3 (Excel Spreadsheet)**

**Description:** Copy number profile for the three patients with *EGFR^19Del^* (P1, P6 and P10).

**File name: Supplementary Data 4 (Excel Spreadsheet**)

**Description:** List of DEGs between 2-NBDG^high^ phenotype and C12R^high^ phenotype shared by all 5 patients.

**File name: Supplementary Data 5 (Excel Spreadsheet)**

**Description:** List of Enrichr results against five public databases.

**File name: Supplementary Data 6 (Excel Spreadsheet)**

**Description:** GSEA for the MSigDB Hallmark and C2 gene sets.
