# Supplementary material for: Liquid biopsy-based single-cell metabolic phenotyping of lung cancer patients for informative diagnostics
Source: Nat Commun. 2019 Aug 26;10:3856. doi: 10.1038/s41467-019-11808-3 (PMC6710267; doi:10.1038/s41467-019-11808-3)
Supplement: Supplementary file 9 — Reporting Summary [file 41467_2019_11808_MOESM9_ESM.pdf]

nature research

Corresponding author(s): Wei Wei, Qihui Shi, Shun Lu

Reporting Summary

Nature Research wishes to improve the reproducibility of the work that we publish. This form provides structure for consistency and transparency in reporting. For further information on Nature Research policies, see Authors & Referees and the Editorial Policy Checklist.

Statistical parameters

When statistical analyses are reported, confirm that the following items are present in the relevant location (e.g. figure legend, table legend, main text, or Methods section).

n/a	Confirmed	
☐	☒	The exact sample size (*n*) for each experimental group/condition, given as a discrete number and unit of measurement
☐	☒	An indication of whether measurements were taken from distinct samples or whether the same sample was measured repeatedly
☐	☒	The statistical test(s) used AND whether they are one- or two-sided<br>*Only common tests should be described solely by name; describe more complex techniques in the Methods section.*
☐	☒	A description of all covariates tested
☐	☒	A description of any assumptions or corrections, such as tests of normality and adjustment for multiple comparisons
☐	☒	A full description of the statistics including central tendency (e.g. means) or other basic estimates (e.g. regression coefficient) AND variation (e.g. standard deviation) or associated estimates of uncertainty (e.g. confidence intervals)
☐	☒	For null hypothesis testing, the test statistic (e.g. *F*, *t*, *r*) with confidence intervals, effect sizes, degrees of freedom and *P* value noted<br>*Give P values as exact values whenever suitable.*
☒	☐	For Bayesian analysis, information on the choice of priors and Markov chain Monte Carlo settings
☒	☐	For hierarchical and complex designs, identification of the appropriate level for tests and full reporting of outcomes
☐	☒	Estimates of effect sizes (e.g. Cohen's *d*, Pearson's *r*), indicating how they were calculated
☐	☒	Clearly defined error bars<br>*State explicitly what error bars represent (e.g. SD, SE, CI)*

Our web collection on statistics for biologists may be useful.

Software and code

Policy information about availability of computer code

Data collection	The following software was used for data collection: BD FACSDiva Software Version 6.2
Data analysis	The following software was used for data analysis: BD FlowJo (version 7.6), GraphPad Prism v7, Microsoft Excel 2010, Origin8 Pro SR4. The packages and pipelines used for analyzing sequencing data have been described in detail in the Methods section.

For manuscripts utilizing custom algorithms or software that are central to the research but not yet described in published literature, software must be made available to editors/reviewers upon request. We strongly encourage code deposition in a community repository (e.g. GitHub). See the Nature Research guidelines for submitting code & software for further information.

Data

Policy information about availability of data

All manuscripts must include a data availability statement. This statement should provide the following information, where applicable:

- Accession codes, unique identifiers, or web links for publicly available datasets
- A list of figures that have associated raw data
- A description of any restrictions on data availability

Sequence raw data have been deposited and are publicly available in the Sequence Read Archive (BioProject accession PRJNA482262). The processed data have been included in Supplementary Data 1-6

Field-specific reporting

Please select the best fit for your research. If you are not sure, read the appropriate sections before making your selection.

☒ Life sciences ☐ Behavioural & social sciences ☐ Ecological, evolutionary & environmental sciences

For a reference copy of the document with all sections, see nature.com/authors/policies/ReportingSummary-flat.pdf

Life sciences study design

All studies must disclose on these points even when the disclosure is negative.

Sample size	The sample size was based upon the number of pleural effusion samples available from stage IV lung adenocarcinoma patients between April 2017 and January 2019. Due to the exploratory nature of the study, it is not appropriate to pre-specify the sample size. Samples were acquired and data analyzed prospectively. We totally acquired 32 patient samples that covered prevalent molecular subtypes of lung adenocarcinoma (as outlined in Table 1) with sufficient samples in each metabolic phenotype for making statistically significant comparisons.
Data exclusions	No data were excluded from the metabolic phenotyping analysis.
Replication	All cell line experiments were reproduced using biological replicates two or more times using the same experimental approach. All attempts to replicate were successful. Due to limited amount of patient samples and the nature of the metabolic assays that require fresh patient samples to be analyzed in a timely fashion, we were unable to perform biological replicates for patient samples.
Randomization	Randomization is not applicable.
Blinding	The results of cytomorphological analysis on pleural effusions, sequencing of cell blocks, and clinical outcomes were blinded to the operators. The evaluation of ECOG score and RECIST were performed in an independent and blinded manner.

Reporting for specific materials, systems and methods

Materials & experimental systems

n/a	Involved in the study
☒	☐ Unique biological materials
☐	☒ Antibodies
☐	☒ Eukaryotic cell lines
☒	☐ Palaeontology
☒	☐ Animals and other organisms
☐	☒ Human research participants

Methods

n/a	Involved in the study
☒	☐ ChIP-seq
☐	☒ Flow cytometry
☒	☐ MRI-based neuroimaging

Antibodies

Antibodies used	Allophycocyanin (APC)-conjugated anti-CD45 (clone HI30): Life Technologies (#MHCD4505-4);<br>Anti-AXL (C89E7) Rabbit mAb: Cell Signalging (#8661S);<br>Anti-PD-L1 (clone RR604): Biolynx (#BX00006);<br>Goat anti-Rabbit IgG (H+L) Cross-Adsorbed Secondary Antibody, Alexa Fluor 488: Invitrogen (#A-11008)
Validation	The antibodies were commercially available and validated by the manufacturer. APC-CD45 was further validated with PBMC from human blood as a positive control and lung cancer cell line A549 as a negative control. Anti-PD-L1 was validated with a PD-L1-overexpressed cell line provided by the manufacturer and a breast cancer cell line SK-BR-3.

Eukaryotic cell lines

Policy information about cell lines

Cell line source(s)	Human lung adenocarcinoma cell lines A549, NCI-H1650 and NCI-H1975, human colorectal carcinoma cell line HCT116, human osteosarcoma cell lines MG63 and 143B were obtained from the cell bank of Chinese Academy of Sciences.
Authentication	Cell lines were authenticated by DNA short tandem repeat (STR) profiling analysis.

Mycoplasma contamination	All tested negative for mycoplasma contamination
Commonly misidentified lines (See ICLAC register)	No commonly misidentified cell lines were used.

Human research participants

Policy information about studies involving human research participants

Population characteristics	Thirty two lung adenocarcinoma patients with pleural effusion were prospectively included in this study between April 2017 and January 2019. All relevant information was summarized in Table 1 and Supplementary Table 1.
Recruitment	Available and qualified pleural effusion samples from lung adenocarcinoma patients were recruited in this study without bias on treatment history, mutation status or results of cytological analysis. The research materials obtained from patients were only in the form of remnant pleural effusion specimens that are routinely collected for pathological diagnosis and therapeutic purpose. No additional specimens were obtained solely or specifically for the purposes of this study. Pleural effusion samples were collected with written informed consent and in accordance with guidelines and protocols that approved by the Ethics and Scientific Committees of Shanghai Chest Hospital.

Flow Cytometry

Plots

Confirm that:

☒ The axis labels state the marker and fluorochrome used (e.g. CD4-FITC).

☒ The axis scales are clearly visible. Include numbers along axes only for bottom left plot of group (a 'group' is an analysis of identical markers).

☒ All plots are contour plots with outliers or pseudocolor plots.

☒ A numerical value for number of cells or percentage (with statistics) is provided.

Methodology

Sample preparation	Single cell suspensions of A549, H1650, H1975, HCT116, MG63, 143B cell lines were collected following the digestion of 0.25% Trypsin-EDTA for 2-3 min, washing with HBSS and staining with 2-NBDG and C12-Resazurin, and then detected by the instrument. White blood cells were isolated from peripheral blood of healthy donors, stained with CD45-APC antibody and mixed with cell lines, and then labeled with 2-NBDG and C12-Resazuin in the tube.
Instrument	Polychromatic flow cytometry was performed by the BD & LSR Fortessa Cell Analyzer.
Software	BD FACSDiva Software Version 6.2 and FlowJo software version 7.6
Cell population abundance	50,000 events were recorded for each sample
Gating strategy	Single cells were identified by FSC-SSC gate. Cells stained with 2-NBDG and C12-Resazurin (C12R) separately were identified as compensation controls prior the analysis of each sample. Unstained controls were used to determine the real positive signals. For metabolite perturbation experiment in Supplementary Fig. 2c, untreated control was used to establish the gates for metabolite-perturbed samples. For the experiment shown in Supplementary Fig. 7, no gating was required because the experiment was designed to evaluate the correlation between the 2-NBDG and C12R signals.

☒ Tick this box to confirm that a figure exemplifying the gating strategy is provided in the Supplementary Information.
